# Posttraumatic growth in psychosis: Symptoms, meaning, and coping

**DOI:** 10.1192/j.eurpsy.2023.1057

**Published:** 2023-07-19

**Authors:** Y. Mazor, M. Gelkopf, D. Roe

**Affiliations:** 1 Department of community mental health, University of Haifa, Haifa; 2 Ono Academic College, Kiryat Ono; 3 School of social work and welfare, Hebrew university of Jerusalem, Jerusalem, Israel

## Abstract

**Introduction:**

Research suggested that psychosis and mental illness-related experiences can be extremely traumatic, and that psychosis could theoretically also lead to posttraumatic growth (Mazor et al., 2019; PTG). The promotion of PTG may contribute to the treatment of people who experienced massive traumas such as people with severe mental illness (SMI). Psychotic symptoms are a common feature of SMI, and individuals who have experienced psychosis are also more likely to have been exposed to trauma and are more vulnerable to developing posttraumatic symptomatology (Ng et al., 2021). Negative symptoms such as amotivation could also contribute to the traumatic experiencing of psychosis (Mazor et al., 2016). Amotivation is specifically relevant to the possible traumatic sequelae of psychosis (Mueser et al., 2010). Alongside the adverse consequences of psychopathological symptoms is the unique outcome of coping with adversity (Tedeschi & Calhoun, 1995)- PTG. Two important factors that contribute to PTG is coping self-efficacy (CSE), and meaning making (Mazor et al., 2018). We investigated the possibility of PTG in individuals with SMI, through the mediating effect of CSE and meaning making.

**Objectives:**

Recent research has shown high rates of exposure to trauma among people with SMI, and that psychosis and mental illness-related experiences can be extremely traumatic. While some develop PTSD, it has been noted that some may also experience PTG. However, few studies have examined PTG in this population.

**Methods:**

121 participants were recruited from community mental health centers and administered trauma and psychiatric questionnaires. Study protocol was approved by the University of Haifa ethics board.

**Results:**

High levels of traumatic exposure were found in the sample. Furthermore, we found that people who endured psychosis can experience PTG, which was mediated by meaning making (MLQ) and CSE. Psychotic symptoms were found to be a major obstacle for PTG, whereas negative symptoms were found to have the potential to lead to PTG when mediated by meaning making and CSE.

*Mediation analyses for the dimensions of PANSS, MLQ total, CSE total, and PTGI total (N=121)*

**Table:** 

Dependent Variable (DV)	Independent variable (IV)	Mediator	IV to mediator B (SE)	Mediator to DV B (SE)	Mediation effect B (SE)	Z
PTGI total	PANSS Positive symptoms total	MLQ total	-0.46 (0.26)	0.99*** (0.10)	-0.46 (0.27)	1.71
		CSE total		-2.08 (0.89)	0.28*** (0.03)	-0.58 (0.28)
	PANSS Negative symptoms total	MLQ total	-0.64*** (.17)	0.93*** (0.11)	-0.60 (0.17)	3.62***
		-2.06*** (0.58)	0.26*** (0.03)	-0.53 (0.17)	3.13**	
	PANSS General psychopathology total	MLQ total	-0.64*** (.12)	0.96*** (0.11)	-0.61 (0.13)	4.77***
		-2.32*** (0.43)	0.27*** (0.03)	-0.62 (0.15)	4.08***	

*Note.*

** p<.01

*** p<.001

**Conclusions:**

The portrayed research provided preliminary evidence for the potential role of meaning making and CSE as mediators of PTG in the clinical, highly traumatized population of people with SMI who have experienced psychosis.

**Disclosure of Interest:**

None Declared

